# Estrogen receptor α K303R mutation reorganizes its binding to forkhead box protein A1 regions and induces chromatin opening

**DOI:** 10.1007/s11033-022-08089-3

**Published:** 2022-11-27

**Authors:** Tomoyoshi Nakadai, Liying Yang, Kohei Kumegawa, Reo Maruyama

**Affiliations:** 1 Project for Cancer Epigenomics, Cancer Institute, Japanese Foundation for Cancer Research, 3-8-31, Ariake, Koto-Ku, Tokyo, 135-8550 Japan; 2 Cancer Cell Diversity Project, NEXT-Ganken Program, Japanese Foundation for Cancer Research, Tokyo, Japan

**Keywords:** ERα, Breast cancer, K303R, MDA-MB-453, FoxA1, NCOA2

## Abstract

**Background:**

Estrogen receptor alpha (ERα) is a frequently mutated gene in breast cancer (BC). While many studies have investigated molecular dysregulation by hotspot mutations at Y537 and D538, which exhibit an estrogen-independent constitutively active phenotype, the functional abnormalities of other mutations remain obscure. The K303R mutation in primary invasive BC has been implicated with endocrine resistance, tumor size, and lymph node positivity. However, the impact of the K303R mutation on the cell epigenome is yet unknown.

**Methods and results:**

We introduced the K303R ERα mutant in ERα-negative MDA-MB-453 cells to monitor ERα-dependent transactivation and to perform epigenomic analyses. ATAC-seq and ChIP-Seq analyses indicated that both wild-type (WT) and the K303R mutant associated with Forkhead box (Fox) protein family motif regions at similar rates, even without an ERα-binding sequence, but only the K303R mutant induced chromatin opening at those regions. Biochemical analyses demonstrated that the WT and the K303R mutant can be tethered on DNA by FoxA1 indirectly, but only the K303R/FoxA1/DNA complex can induce associations with the nuclear receptor cofactor 2 (NCOA2).

**Conclusions:**

These findings suggest that the K303R mutant induces chromatin opening at the Fox binding region through the FoxA1-dependent associations of the K303R mutant to NCOA2 and then probably disrupts the regulation of Fox-target genes, resulting in K303R-related BC events.

**Supplementary Information:**

The online version contains supplementary material available at 10.1007/s11033-022-08089-3.

## Introduction

About two-thirds of breast cancer (BC) cases are positive for estrogen receptor alpha (ERα) [1]. ERα is a member of the nuclear hormone receptor (NHR) superfamily. Estrogen binds to the C-terminal ligand-binding domain (LBD) of ERα, and then estrogen-bound ERα binds to an estrogen response element (ERE) of genomic DNA regions. DNA-bound ERα recruits multiple transcription cofactors to activate transcription [2]. In ~ 20% of patients with metastatic BC, the endocrine treatment causes missense *ESR1* mutations mainly at the Y537 and D538 residues of the LBD [3], which constitutively activate transcription without estrogen, resulting in the “constitutively active” phenotype that is insensitive to anti-estrogen drugs [3].

Other various *ESR1* mutations have also been detected in clinical BC specimens but at lower frequencies than the Y537 and D538 mutations [4]. Among them, the K303R mutation is found in 5%–10% of primary invasive BC cases, but can only be identified by an accurate detection method, known as single-strand conformation polymorphism, rather than conventional sequencing methods, although the exact occurrence rate of the K303R mutation remains to be elucidated [5]. The K303R mutation induces S305 phosphorylation, which has been implicated in resistance to tamoxifen and aromatase inhibitors, and enhanced transactivation ability [6–8]. In addition, the K303R mutation alters the affinity of the NHR coactivator NCOAs to ERα [9] and induces ERE-derived reporter gene transcription and cellular proliferation [10, 11]. The K303R mutation is also associated with a first-degree family history of BC, larger tumor size, and axillary lymph node positivity [12, 13]. Therefore, the K303R mutation is thought to be related to the development of BC. However, the global effect of the K303R mutation on the epigenome/cistrome remains unclear. Therefore, further investigations are needed to elucidate the mechanisms underlying the abnormal functions of the K303R mutation in BC.

To analyze the functional abnormality of the ERα mutation, the ectopic ERα mutant was assessed in ERα-expression-negative MDA-MB-453 cells using the assay for transposase-accessible chromatin using sequencing (ATAC-seq) and chromatin immunoprecipitation combined with sequencing (ChIP-Seq). The results revealed that K303R-specific regions harboring both features of chromatin accessibility and ERα-binding were enriched with Forkhead box (Fox) protein family binding motifs, but without an ERE. While the K303R mutant and WT ERα bound to the Fox motif regions without an ERE at a similar rate, the K303R mutant but not the WT strongly induced chromatin opening of these regions. Subsequent biochemical analyses showed that DNA-bound FoxA1 indirectly recruits both the WT and K303R mutant forms of ERα to DNA, while only the K303R mutant specifically recruited the histone-modifying hub nuclear receptor coactivator 2 (NCOA2) to DNA.

## Materials and methods

### Plasmids, primers, antibodies, qPCR, cells, proteins, and viruses

Detailed information for plasmids, primers, antibodies, qPCR, cells, proteins, and viruses used in the present study was described in Supplemental Materials and Methods.

### Luciferase assay

Luciferase assays were performed as described [14] with modifications as described in Supplemental Materials and Methods.

### Establishment of MDA-MB-453 clones expressing ERα

MDA-MB-453 cells were stably transduced with infection of lentivirus prepared from pAiLV-FH-ERα plasmids (see above) or empty backbone vector, in the presence of 8 µg/ml Polybrene (Sigma). After one week of culture, GFP-positive cells were single-cell-sorted and expanded. The expression of ectopic FH-ERα was induced by 2 µg/ml doxycycline (Dox) (Sigma), and checked by western blotting.

### ATAC-seq, ChIP-seq, and data analysis

Detailed protocols and data analysis for ATAC-seq and ChIP-seq were described in Supplemental Materials and Methods. The raw NGS data was uploaded to GSE191065.

### Immobilized template assay

Immobilized template assays (ITA) [15] were performed with modifications as described in Supplemental Materials and Methods.

## Results

### Ectopic ERα activates transcription via the EREs in various BC cell lines

**Fig. 1:**
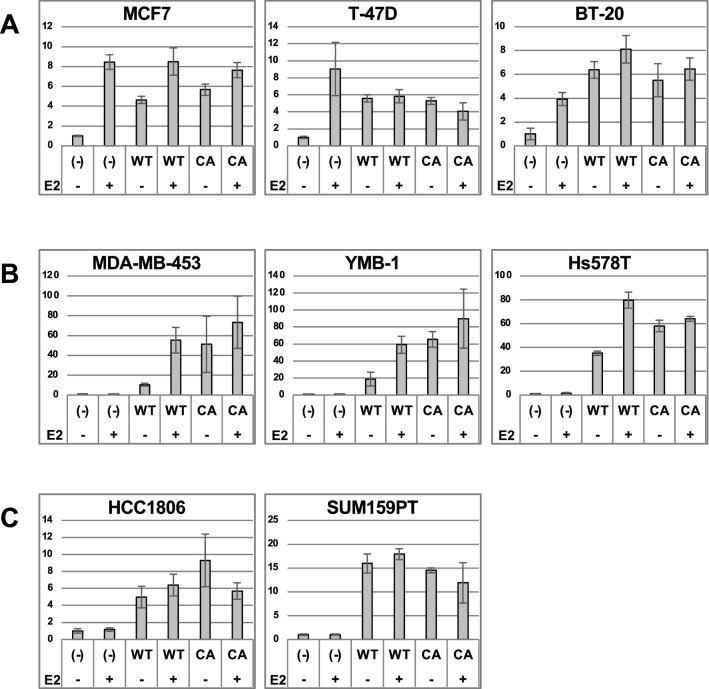
The luciferase assay with various BC cell lines. The luciferase assay was performed with WT or D538G mutant (CA, constitutive active)-expressing plasmids with or without E2 in various BC cell lines. The firefly luciferase signals normalized with control *Renilla* luciferase signals in three independent assays are plotted as fold-increases relative to the ERα(−)/E2(−) condition

To explore the novel abnormality of the K303R mutant under physiological conditions, we planned to express the ectopic K303R mutant in BC cell lines, and then analyze the effects on the epigenome/cistrome. To efficiently detect the specific function of this mutant, the model cell line should satisfy the following criteria: (i) no endogenous ERα activity, (ii) detectable ectopic ERα-dependent activity, (iii) estrogen-dependent activity, and (iv) mutant-specific activity (i.e., the constitutively active phenotype). The various BC cell lines (Fig. S1) were subjected to the luciferase assay with a reporter gene driven by EREs. The results showed that several cell lines exhibited luciferase gene expression with varied responsivity to ERα and E2, while some cell lines were not responsive (Figs. 1, S1). The first group (Type A) expressed the reporter gene even without WT ectopic ERα in the presence of E2, indicating E2-bound endogenous ERα activates ERE-driven transcription because the cells express endogenous ERα at high levels (Fig. 1A). The second group (Type B) exhibited ectopic ERα-dependent transcription in an E2-dependent manner and E2-independent transcription by the constitutive active D538G mutant (Fig. 1B). The third group (Type C) exhibited ectopic ERα-dependent transcription but were not responsive to E2, indicating the lack of the constitutively active phenotype (Fig. 1C). The differences in the responsiveness among the tested cell lines, including complete lack of ERE activity (Fig. S1), were not correlated to expression levels of *ESR1/ERBB2/PGR*, molecular classification, or important gene mutations in BC (Fig. S1). The cell lines in the type B group met the four criteria described above. Especially, among them, MDA-MB-453 cells, which were shown to express *ESR1* very slightly and *ERBB2* strongly by our qPCR analysis as reported previously (Fig. S1), exhibited the clearest E2-dependent activity, and thus, were chosen for analyses of the K303R mutant.

### The abnormal accessible chromatin region differs between the ERα mutations K303R and Y537S

**Fig. 2:**
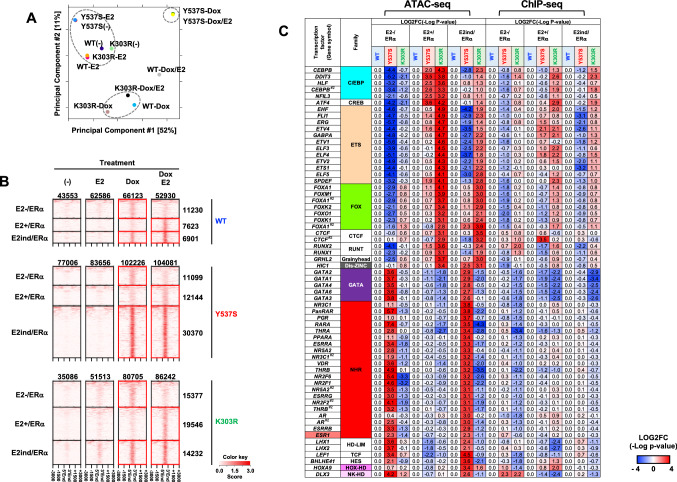
ATAC-seq and ChIP-seq analysis of the established MDA-MB-453 clones. **A** PCA of ATAC-seq peak data. Specific groups are rounded by dashed line circles. **B** Heatmap showing the signal intensity of ATAC-seq peaks in each condition-specific group (red boxes) and those corresponding genomic regions in other groups of WT/MDA-MB-453, Y537S/MDA-MB-453, and K303R/MDA-MB-453. Peak intensities are plotted in a ± 3 kb window from summits. **C** Comparison of TFBM enrichment among the condition-specific ATAC-seq (left half) and ChIP-seq (right half) peak groups of each clone. TFBMs’ enrichment score (− Log(*p*-value) of Y537S/MDA-MB-453 and K303R/MDA-MB-453 were compared to that of WT/MDA-MB-453 among each group and remarkably enriched TFBMs in the mutant clones were shown with ERE (see “Materials and methods” section for the detailed analysis). (Colour figure online)

Next, MDA-MB-453 cells were established with inducible gene expression of WT ERα (WT/MDA-MB-453) and the K303R ERα mutant (K303R/MDA-MB-453), and also Y537S ERα mutant (Y537S/MDA-MB-453) as a control to evaluate whether the MDA-MB-453 system was proper to detect a functional abnormality. Each of the clones, which express a comparable level of ectopic ERα (Fig. S2A), was treated with E2 to induce estrogen sensitivity, with or without Dox to express ERα, and then subjected to ATAC-seq analysis. Differential binding affinity analysis (DBA) and principal component analysis (PCA) were performed to compare the similarities of genomic accessible regions among the clones (Fig. 2A, S2B). Under both Dox(−)/E2(+) and DOX(−)/E2(−) conditions, the accessible chromatin landscapes were similar among all three clones, indicating comparable background phenotypes with limited effects of E2. The accessible chromatin landscapes of Y537S/MDA-MB-453 under the Dox(+)/E2(−) and Dox(+)/E2(+) conditions were very similar to those of WT/MDA-MB-453 under the Dox(+)/E2(+) condition. These results were clearly due to the constitutively active phenotype of Y537S and confirmed that the MDA-MB-453 system is a convenient biological system to explore abnormalities specifically associated with ERα mutations. On the other hand, the accessible chromatin landscapes of K303R/MDA-MB-453 under the DOX(+)/E2(−) and DOX(+)/E2(+) conditions were largely distinct from that of Y537S/MDA-MB-453, and were similar to those of WT/MDA-MB-453 under the DOX(+)/E2(−) condition (Fig. 2A and S2B), indicating that the K303R mutant is relatively insensitive to E2.

Enrichment analysis of transcription factor binding motifs (TFBMs) at open regions specifically detected under each condition (nontreated, E2(+), Dox(+), or DOX(+)/E2(+)) (Fig. S2C, D) was conducted using the ATAC-seq data of WT/MDA-MB-453 to identify the system allowing similar functions of the WT ERα-positive phenotypes. The results showed that the palindromic ERE was greatly enriched in DOX(+)/E2(+)-specific groups, indicating that ERα can open the ERE region (Fig. S2E). Also, the binding motif of nuclear receptor subfamily 2 group F (NR2F), which is reported to interact with ERα of luminal-type BC cells [16, 17], was enriched in DOX(+)/E2(+)-specific groups. Moreover, the AP-1 family binding motifs were significantly enriched in the Dox(+)/E2(−)-groups, reminiscent of the non-classical function of ERα in ERα-positive cells, i.e., recruitment of ERα by the AP-1 family [18]. Overall, the results of TFBM enrichment analysis at accessible chromatin regions of WT/MDA-MB-453 indicated that the function of WT ERα was similar in MDA-MB-453 and conventional ERα-positive cells, which again supports the application of the MDA-MB-453 system for functional analysis of ERα.

Next, the condition-specific accessible regions of each clone, which were only detected under the *Dox(+)/E2(−)*, *Dox(+)/E2(+)*, and both of the *Dox(+)/E2(−) and Dox(+)/E2(+)* conditions, were classified into the following groups (Fig. 2B): E2-free ERα-dependent (E2 − /ERα), E2-bound ERα-dependent (E2+/ERα), and ERα-dependent/E2-independent (E2ind/ERα). It should be noted that the peaks in E2−/ERα were detected ONLY in the absence of E2 and were close in the presence of E2. Then, the differentially enriched TFBMs of Y537S/MDA-MB-453 and K303R/MDA-MB-453 were compared against those of WT/MDA-MB-453 under each condition (Fig. 2C, left half). The results showed that the ERE was significantly increased in the E2−/ERα and E2ind/ERα groups of Y537S/MDA-MB-453, which indicated that the ERE elements were opened by Y537S in an E2-independent manner. On the other hand, in the E2+/ERα groups, TFBM enrichment was similar between Y537S/MDA-MB-453 and WT/MDA-MB-453, confirming that the constitutively active phenotype of Y537S binds to the ERE in an E2-independent manner [19]. Interestingly, together with the ERE, the binding motifs of the GATA and NHR families were significantly enriched in the E2−/ERα and E2ind/ERα groups of Y537S/MDA-MB-453, in agreement with previous reports of the functional relationship between these transcription factors and ERα [16, 17, 20–22]. E2-independent enrichment of the TFBMs of Y537S/MDA-MB-453 reproduced the constitutive active abnormality of the Y537S mutant even in MDA-MB-453 cells.

Significant differences were observed in the E2+/ERα and E2ind/ERα groups and less in the E2−/ERα group of K303R/MDA-MB-453. The binding motifs of BC-related pioneer factor Fox family [23] were significantly increased in K303R/MDA-MB-453. TFBMs were also enriched for RUNX1/2, GRHL2, and the ETS family which have been implicated in the progression of poor prognosis of BC [24–26]. Taken together, TFBMs enriched at accessible chromatin regions of the K303R mutant significantly differed from those of the WT and constitutive active mutant Y537S.

### K303R differentially regulates the Fox motif regions and induces chromatin opening

Next, ChIP-seq analysis with an anti-ERα antibody was performed to investigate the direct relationship of ERα with the formation of accessible chromatin regions. Comparisons of the ChIP-seq and ATAC-seq data to identify differential TFBMs enrichment in each clone revealed that the differences of the ChIP-seq data were relatively smaller than those of the ATAC-seq data (Fig. 2C, right half). For example, in K303R/MDA-MB-453, ETS and Fox motifs detected by ATAC-seq analysis were only slightly enriched by ChIP-seq analysis. These data indicate that the ERα-binding profiles of the genomic regions were moderately affected by the mutations, while the accessible chromatin regions were largely impacted.

**Fig. 3:**
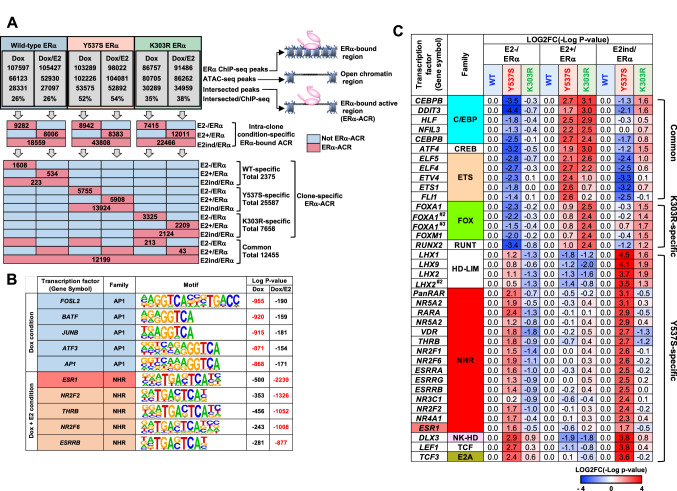
ERα-ACR analysis. **A** Representation of each specific group. The overlapping ChIP-seq and ATAC-seq peaks were selected to define ERα-ACR and then classified into intra-clone condition-specific and clone-specific groups. The peak numbers are also indicated. **B** Top 5 enriched TFBMs in WT ERα-ACR of the Dox(+)/E2(−) and Dox(+)/E2(+)-specific groups. Log(*p*-value) of the top 5 enriched TFBMs in each group and those corresponding values in other groups are represented with a motif logo. **C** Comparison of TFBM enrichment among the condition-specific WT, Y537S, and K303R ERα-ACR groups. Remarkably enriched TFBMs were shown as in Fig. 2C. (Colour figure online)

Therefore, the overlapping ChIP-seq and ATAC-seq peaks were selected to define the ERα-bound active chromatin region (ERα–ACR), where chromatin regions that were bound by ERα were open chromatin structure which was considered active genomic locus related to gene activation (Fig. 3A, top). WT ERα-ACR revealed enrichment of motifs for the AP-1 and the NHR family (i.e., ERα and NR2Fs) under the Dox(+)/E2(−) and Dox(+)/E2(+) conditions, respectively, which were similar to those from ATAC-seq analysis (Fig. 3B and S2E). Next, ERα-ACR was classified into each condition-specific group within clones (E2 − /ERα, E2 + /ERα, and E2ind/ERα) (Fig. 3A, middle) and enriched TFBMs were analyzed (Fig. 3C). The results were significantly different from those of the ATAC-seq analysis. First, the GATA and GRHL2 motifs were absent from Y537S/MDA-MB-453 and K303R/MDA-MB-453 respectively, indicating that these TFBMs were not directly regulated by the binding of mutant ERα, but might be indirectly activated downstream of the mutants. On the other hand, the ERα and NHR motifs of Y537S/MDA-MB-453, and the Fox and RUNX2 motifs of K303R/MDA-MB-453 were clearly reproduced, indicating that all are directly regulated and activated by binding to the mutants.

**Fig. 4:**
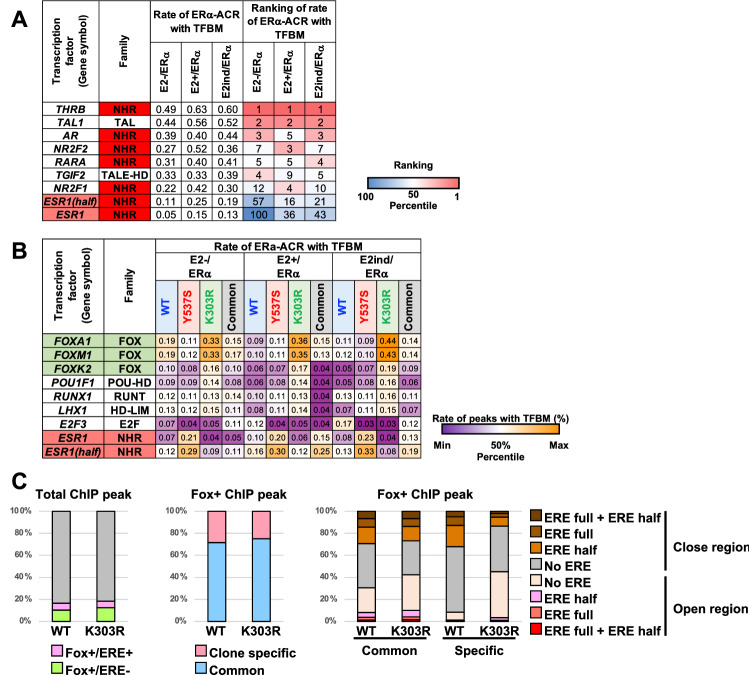
Detailed profiling of K303R ERα-ACR. **A** Rate of ERα-ACR having each TFBM in the common groups. TFBMs with the top 5 rates and ERE in each common ERα-ACR (Fig. 3A, bottom) were represented with the rate. Highly enriched TFBMs (− Log(*p*-value) ≤ 10^–20^) in raw ERα-ACR in any clones and conditions were selected. **B** Comparison of the rate of clone-specific ERα-ACR having each TFBM. The rate of clone-specific ERα-ACR having each TFBM was analyzed. The rates of TFBMs that had a large difference among clones (Max/Min ≥ 3, Max rate in any conditions ≥ 15%) were represented with ERE. **C** Detailed analysis of FoxA1 + ERα-ChIP peaks of WT/MDA-MB-453 and K303R/MDA-MB-453 clones. The rate of ERE motifs in FoxA1 + ERα-ChIP peaks of each clone (left), and the overlap rate among them (middle) were represented. The common and clone-specific FoxA1 + ERα-ChIP peaks were further analyzed for the presence of ERE (full and half sites) and the chromatin opening by comparing with ATAC-seq data, and then each rate is represented (right). (Colour figure online)

To better clarify the mutant-specific phenotype, the clone-specific and common ERα-ACR across all clones were selected and each TFBM at ERα-ACR was counted (Fig. 3A, bottom). The results showed that the top five enriched TFBMs of the common ERα-ACR included various members of the NHR family (i.e., NR2Fs, THRB, AR, and RARA) (Fig. 4A). Surprisingly, the proportion of ERE-related peaks was not notably high in the common groups, indicating that ERα binds to various genomic loci in an ERE-independent manner, while the proportions of ERE half and full sites were significantly enriched by E2 treatment from 11 to 25% and from 5 to 15%, respectively. Next, the proportions of TFBMs of each clone-specific (WT, Y537S, and K303R) ERα-ACR were compared with those of the corresponding common ERα-ACR, and remarkably enriched TFBMs under any condition were extracted (Fig. 4B). The results showed that WT-specific TFBMs were absent. Y537S-specific TFBMs were not obvious, except for the EREs in the E2−/ERα and E2ind/ERα groups, which represented the E2-independent constitutively active phenotype of the Y537S mutant. On the other hand, Fox motifs were the most differentially enriched TFBMs of K303R-specific ERα-ACR under all three conditions. These results confirmed that the functional abnormality of the K303R mutant was likely exhibited through the Fox family.

Next, the raw ChIP-seq and ERα-ACR data were compared to analyze the detailed features of the Fox-related abnormality of the K303R mutant. The results showed that there was no significant difference in the proportion of ERα ChIP-seq peaks with the FoxA1 motif (FoxA1 + ERα-ChIP peak) between the WT and K303R mutant (16.5 vs. 18.5%, respectively, Fig. 4C, left), which was similar to the proportion in a previous report [27]. Interestingly, about two-thirds of the FoxA1 + ERα-ChIP peaks of the WT and K303R mutant did not harbor an ERE (63.6 and 68.1%, respectively), indicating that these ERα associations were independent of the DNA binding ability of ERα and both the WT and K303R mutant forms of ERα were tethered to the genomic region by FoxA1. Up to 80% of the FoxA1 + ERα-ChIP peaks were shared by the WT and K303R mutant, indicating that the K303R mutant partially rearranged the FoxA1 + ERα-ChIP peak (Fig. 4C, middle). Next, the chromatin opening of these regions was analyzed by comparing the FoxA1 + ERα-ChIP peaks with the ERα-ACR data. The results showed that the FoxA1 + ERα-ChIP peaks were relatively open in K303R/MDA-MB-453 as compared to WT/MDA-MB-453, regardless of the existence of ERE motifs, and this difference was more prominent in the K303R mutant- and the WT-specific FoxA1 + ChIP peaks (45.2% vs. 8.5%, respectively, Fig. 4C, right). Similar results were also observed for FoxM1 and Fox K2 (data not shown). Taken together, these results showed that the K303R mutant rearranged the ERα distribution in Fox regions and induced chromatin accessibility of newly generated K303R-bound Fox regions.

### Mechanism of K303R-induced chromatin opening and ERα tethering by FoxA1

**Fig. 5:**
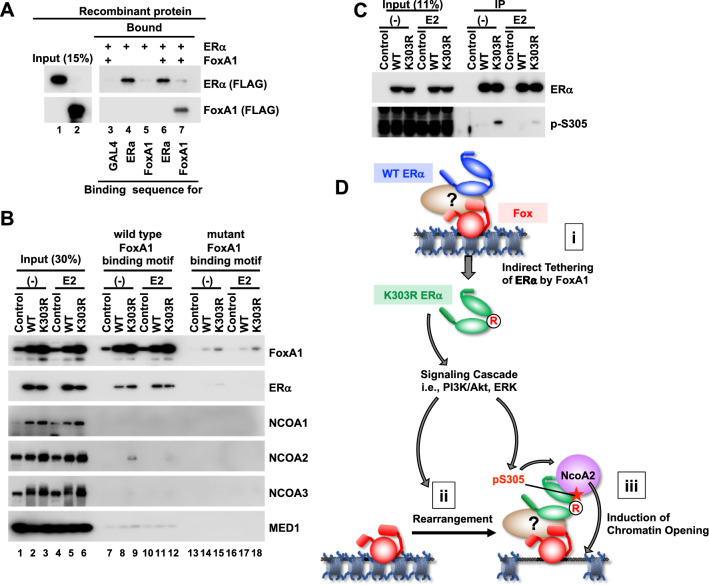
Biochemical analysis of the K303R mutant. **A** The ITA with recombinant ERα and FoxA1 to detect DNA association of ERα and FoxA1. DNA templates having each binding sequence and recombinant proteins were mixed and precipitated fractions were analyzed by western blotting. **B** The ITA with the nuclear extract from control MDA-MB-453, WT/MDA-MB-453, and K303R/MDA-MB-453. The ITA was performed as in **A** with the nuclear extracts from each clone. **C** S305 phosphorylation state of WT and K303R in cells. ERα was immunoprecipitated from the nuclear extract of control MDA-MB-453, WT/MDA-MB-453, and K303R/MDA-MB-453, and ERα and phosphorylated S305 were detected by western blotting. **D** The model for the functional abnormalities of the K303R mutant. The significant rates of both WT ERα and K303R mutant are indirectly tethered by Fox to the genomic region (i). The K303R mutation rearranged Fox-tethering ERα (ii) and the chromatin of the newly generated Fox-tethering K303R ERα was open with NCOA2 association (iii). This K303R-dependent functional alteration of ERα is probably induced downstream of the K303R-activated signaling cascade and S305 phosphorylation. (Colour figure online)

Biochemical analysis was conducted to elucidate the molecular mechanism underlying the tethering of ERα by FoxA1 and K303R-specific induction of chromatin opening. First, ITA was used to assess the direct binding ability between FoxA1 and ERα. Recombinant FoxA1 and ERα bound to the DNA template via unique binding sequences (Figs. S3, 5A, lanes 4, 6, and 7). When FoxA1 and ERα were mixed with the DNA template of the FoxA1 binding sequence, ERα was not recruited to the template (Fig. 5A, lane 7), indicating that FoxA1 did not directly tether ERα. Next, The ITA was performed using nuclear extract derived from WT/MDA-MB-453 and K303R/MDA-MB-453. The results showed that FoxA1 was activated by ectopic ERα expression (Fig. 5B, lane 1–6), possibly by a positive feedback mechanism between ERα and FoxA1 expression [28]. The ITA results showed that the WT and K303R mutant were recruited to the DNA in a FoxA1 binding motif-dependent manner regardless of the presence of E2 (Fig. 5B, lanes 7–12 vs. 13–18), indicating that tethering of ERα by FoxA1 had occurred regardless of the mutation and likely indirectly regulated by an unknown factor(s) in the extracts. To clarify the mechanism underlying K303R-specific chromatin opening, the presence of well-known ERα coactivators (i.e., NCOAs and Mediator complex) [29] in the FoxA1/ERα complex on the DNA template was assessed. Interestingly, the expression of all NCOAs was induced by ectopic expression of the WT and K303R mutant, as observed for FoxA1 (Fig. 5B, lanes 1–6). Among the coactivators, the K303R mutant specifically induced the association of NCOA2 to the DNA template in a FoxA1-dependent and an E2-independent manner (Fig. 5B, lanes 9 and 12). On the other hand, immunoprecipitation of ERα from nuclear extracts of the clones did not show the increased association of NCOA2 to K303R (data not shown), indicating the K303R mutation did not simply induce NCOA2 association to ERα but probably K303R/FoxA1/DNA complex cooperatively recruited NCOA2 onto DNA. The K303R mutant induced S305 phosphorylation in K303R/MDA-MB-453 (Fig. 5C) as reported [6, 7]. These results indicate that WT ERα was fundamentally tethered by FoxA1 to the DNA indirectly and the K303R mutant induced NCOA2 association to the ERα/FoxA1/DNA complex.

## Discussion

ATAC-seq and ChIP-seq analyses followed by molecular biochemical analysis showed that K303R-specific functional abnormalities were related to the rearrangement of ERα-bound FoxA1 motif regions and the accessibility of these regions probably via K303R-induced S305 phosphorylation and recruitment of NCOA2 (Fig. 5D, ii, iii). In addition, FoxA1 was revealed to tether ERα to genomic regions regardless of ERα mutation (Fig. 5D, i).

### ERα mutant abnormality analysis with MDA-MB-453 cells

MDA-MB-453 in addition to YMB-1 and Hs578T cells facilitated analysis of ectopic ERα function as each of these cell lines exhibited ectopic ERα and E2-responsive luciferase gene expression (Fig. 1B). Moreover, some cell lines were insensitive to E2 and exhibited no ERE activity (Fig. S1). Further analyses should be conducted to identify what underlies the differences in ERα-responsiveness among the cell lines. Because the expression patterns of various ERα cofactors varied among the tested cell lines (Fig. S4), the cooperative and exclusive activities might be responsible for differences in responsiveness to ectopic ERα.

MDA-MB-453 cells were selected for analyses of the Y537S and K303R mutants with WT ERα. The results revealed that in the WT ERα cistrome, ERα is recruited to the AP-1 region without E2 and to the NHR region with E2. Moreover, the Y537S mutant conveyed constitutive activation of the cistrome. The enhanceosome, consisting of ERα, FoxA1, and GATA3, was required for the BC-associated effects of ERα [30] and MDA-MB-453 cells highly express FoxA1 and GATA3 comparable to the luminal-type MCF7 and T-47D cells (Fig. S4). Therefore, the WT and Y537S mutant probably can directly or indirectly establish the WT and constitutively active cistrome, respectively, even in MDA-MB-453 cells, as in luminal-type cell lines, indicating that ERα-negative MDA-MB-453 cells are a useful model to analyze the functions of ectopic ERα.

### The K303R mutant rearranges ERα-bound regions with Fox family binding motifs

The motifs of the Fox family FoxA1, FoxM1, and FoxK2 are significantly expressed in MDA-MB-453 cells (Fig. S5) and enriched in the ERα-ACR of K303R/MDA-MB-453 (Fig. 4). The transcription factor FoxA1 functions as a pioneer factor that binds to and opens the compacted chromatin, and induces sequential bindings of ERα, which are required for ERα-target gene regulation in BC cell lines [28, 31]. FoxM1 has been associated with the onset and poor prognosis of BC [32] while FoxK2 suppresses the proliferation of BC cells by associating with corepressors [33].

The landscape of the ERα-bound FoxA1 motif was partially altered by the K303R mutant (Fig. 5C,D-ii). ERα binding to FoxM1 and FoxK2 was also altered by the K303R mutant (data not shown). Because ChIP-seq analysis revealed that the Fox family shares similar binding motifs (Fig. S6), The Fox family might redundantly function via TFBMs. The K303R mutant is reported to activate the insulin-like growth factor 1 receptor and the downstream PI3K/AKT signaling cascade [6, 34], and potentially the downstream MAPK/ERK pathway in BC cells [35]. These pathways positively regulate FoxA1 and FoxM1 via protein stabilization, nuclear translocation, and transactivation induction mechanisms [36, 37]. These pathways also regulate the nuclear localization of FoxK2 for target gene expression [38]. Therefore, K303R-induced signaling pathways may alter the balance of orchestral Fox family recruitment to Fox motifs, resulting in the rearrangement of the landscape of Fox-tethering ERα (see below) in K303R/MDA-MB-453.

### The K303R mutation increases chromatin accessibility at ERα-bound regions with Fox family binding motifs

Comparisons of the ATAC-seq and ChIP-seq data revealed that the ERα-bound Fox motif regions tended to be more accessible as ERα-ACR in K303R/MDA-MB-453 (Figs. 4C, 5D-iii). Although further analysis is needed to elucidate the molecular mechanism underlying the change in accessibility, S305 phosphorylation is a possible mechanism in K303R/MDA-MB-453 (Fig. 5C). The K303R mutation induces S305 phosphorylation [6], which is related to tamoxifen resistance [39] and enhances the association of the NHR transcription coactivator NCOA1 and ERα [7]. Interestingly, because MDA-MB-453 cells harbor high HER2 expression (Fig. S1) and constitutive active K-RAS mutation G13D [40], K303R-dependent S305 phosphorylation might be further induced by active HER2/K-RAS/MAPK/ERK pathway in the cell. The K303R mutation also induces associations of NCOA1 and NCOA3 with ERα [9]. The NCOA family are recruited to the enhancer by NHR, including ERα, and then induce local nucleosome modification via associations with histone-modifying enzymes [29]. In the present study, among the NCOA family, only NCOA2 was found to significantly associate with the FoxA1/ERα complex on DNA by the K303R mutation in an E2-independent manner. ERα poses two transcription activation domains which are activation function 1 and 2 (AF1 and AF2). The binding of NCOA2 to the N-terminal AF1 domain of ERα is independent of E2 while the association of NCOA2 with the C-terminal AF2 domain of ERα is dependent on E2 [41]. Moreover, because immunoprecipitation of ERα did not reveal an increased association of NCOA2 with the K303R mutant (data not shown), the K303R mutation and following S305 phosphorylation did not simply induce an association with NCOA2. Furthermore, chromatin opening was specifically induced at regions with the Fox motifs, but not global K303R ERα-ACR with other TFBMs in K303R/MDA-MB-453 (Fig. 4). Therefore, FoxA1 may take a part in the recruitment of NCOA2 to AF1 region of K303R phosphorylated at S305 although further analysis is needed to determine how the AF1 region of S305-phosphorylated K303R and FoxA1 induce the recruitment of NCOA2 to the DNA and whether NCOA2 contributes to chromatin accessibility at K303R ERα-ACR in K303R/MDA-MB-453.

### Novel insights in the fundamental molecular activities of ERα–indirect tethering of ERα by Fox

While various Fox factors are generally considered to induce sequential binding of ERα to the ERE [28, 31], other reports have indicated that ERα binds to FoxA1 motifs even without ERE motifs [42]. This study clearly demonstrates that both WT and K303R ERα associated with the Fox motif regions without the ERE (Fig. 4). Therefore, the rate of ERα association with the Fox motif regions is likely regulated not only by the pioneer mechanism but also by a “tethering” mechanism of the Fox as proposed for the non-classical function of ERα via the AP-1 family [18]. Coincidently, the results of the biochemical analysis demonstrated that both WT and K303R ERα were indirectly tethered by FoxA1 through unidentified factors (Fig. 5B,D-i). FoxA1 binds to well-positioned tight chromatin as a pioneer factor and induces sequential binding of other DNA-binding activators for transcription activation [43]. Because the ITA analyzes simple protein–protein bindings on naked DNA-template, the observed tethering of ERα by FoxA1 might happen after FoxA1 binds to chromatin DNA in cells. The present study indicated that ERα may act as a non-DNA-binding coactivator for FoxA1. It should be emphasized that only K303R but not WT ERα increased the chromatin accessibility via NCOA2 recruitment after the tethering by FoxA1 (see above). Further studies are warranted to identify factors that mediate indirect tethering and to determine whether the Fox family FoxM1 and FoxK2 can also tether ERα.

### Relation of K303R mutation and the new findings to breast cancer

FoxA1 overexpression reportedly mediates pro-metastatic enhancer reprogramming and migration of endocrine-resistant BC cells [44]. In the present study, the K303R mutation increased chromatin accessibility at a certain set of Fox motif regions. Although the specific target genes remain unclear, the K303R mutation might mainly modulate the transcriptome in a Fox-dependent manner, which may account for the larger tumor size and axillary lymph node positivity in BC with the K303R mutation [12].

Endocrine treatment generates various genetic mutations, including endocrine-resistant ERα hotspot mutations, in metastatic/recurrent BC [45]. Thus, the continued develop new drugs is necessary to overcome resistance (ClinicalTrials.gov). A large part of ERα is indirectly tethered by Fox via an unknown mediating factor, which presents a potential new therapeutic target to inhibit the FoxA1–ERα axis in ERα+ metastatic/recurrent BC. This study also indicated that K303R-specific chromatin opening might be dependent on S305 phosphorylation and recruitment of NCOA2 (Fig. 5). Because S305 phosphorylation reduces sensitivity to endocrine therapy and induces ligand independency concomitant with stabilization of ERα on the promoter region and increased transactivation ability [7, 46], S305 phosphorylation may induce chromatin opening of Fox motif regions and target gene activation through NCOA2 in endocrine-resistant cells. Moreover, NCOA2 is reportedly required for the proliferation of various BC cell lines and is related to the poor prognosis of BC [47]. Therefore, inactivation or removal of NCOA2 can be a new molecular therapy not only against BC bearing K303R mutation which induces S305 phosphorylation and then NCOA2 recruitment to ERα/Fox but also against endocrine-resistant BC in which elevated S305 phosphorylation may enhance NCOA2 recruitment to ERα/Fox.

## Supplementary Information

Below is the link to the electronic supplementary material.

Supplemental Materials and Methods (PDF 143 kb)

Supplemental Figures (PDF 9973 kb)

Supplemental Table 1 (XLSX 16 kb)

Supplemental Table 2 (XLSX 1266 kb)

Supplemental Table 3 (XLSX 56 kb)

## Abbreviations

ER: Estrogen receptor
BC: Breast cancer
WT: Wild-type
NHR: Nuclear hormone receptor
LBD: Ligand-binding domain
ERE: Estrogen response element
ATAC-seq: The assay for transposase-accessible chromatin using sequencing
ChIP-seq: Chromatin immunoprecipitation combined with sequencing
E2: 17β-Estradiol
Dox: Doxycycline
TFBM: Transcription factor binding motif
NCOA: Nuclear receptor coactivator
