# Supplementary material for: Estrogen receptor α K303R mutation reorganizes its binding to forkhead box protein A1 regions and induces chromatin opening
Source: Mol Biol Rep. 2022 Nov 27;50(2):1209–20. doi: 10.1007/s11033-022-08089-3 (PMC9889408; doi:10.1007/s11033-022-08089-3)
Supplement: Supplementary file 1 — Supplemental Materials and Methods (PDF 143 kb) [file 11033_2022_8089_MOESM1_ESM.pdf]

Molecular Biology Reports

Estrogen receptor α K303R mutation reorganizes its binding to Forkhead box protein A1 regions and induces chromatin opening.

Tomoyoshi Nakadai[1], Liying Yang[1], Kohei Kumegawa[2], Reo Maruyama[1,2]

1 Project for Cancer Epigenomics, Cancer Institute, Japanese Foundation for Cancer Research, Tokyo, Japan.
2 Cancer Cell Diversity Project, NEXT-Ganken Program, Japanese Foundation for Cancer Research, Tokyo, Japan.

Corresponding author's contact information:
Tomoyoshi Nakadai
Project for Cancer Epigenomics
Cancer Institute, Japanese Foundation for Cancer Research
3-8-31, Ariake, Koto-ku, Tokyo, 135-8550, Japan
Tel: +81-3-3520-0111
E-mail: tomoyoshi.nakadai@jfcr.or.jp

Supplemental Materials and Methods

Primers used in the study

Primers for pGL3-GREB1-ERE3-fLuc construction are as follows; forward 5'-GCGCGGATCC**GGTACC**AGTGTGGCAACTGGGTCATTC-3', reverse 5'-GCGC**AAGCTT**CGGCCGCTCCAGTCACCACACGTGTTA-3'. Underline and bold sequences indicate genomic sequence and restriction enzyme sites, respectively.

Primers for RT-qPCR

	Forward	Reverse
ERα	CCTTCTTCAAGAGAAGTATTCAAGG	ATTCCCACTTCGTAGCATTTG
ERBB2	CCCTGACCTGCTGGAAAAG	GGCCGACATTCAGAGTCAAT
PGR	GGATGAGCTTAATGGTGTTTGG	GATTCTTTCATCCGCTGTTCA

Linker and tag sequences, and restriction sites for plasmid construction

1) ERα in pcDNA3.1(-) vector for the effector plasmid of luciferase assay

GGTACCGAGCTCGGATCTGCCACC**ATGGACTACAAAGACCACGACGGCGACTACAAAGACCACGACATAGACTACAAAGACGACGATGACAAACAT**-*ERα*-GGATCC

Underlines, double underline, and bold sequences indicates KpnI/BamH sites, kozak, and triple FLAG-tag sequences, respectively. The position of *ERα* ORF from the 1st methionine to STOP codon is indicated in italic letters.

2) ERα in lentiviral vector pAiLV for the ectopic expression of ERα in MDA-MB-453 cells

CGTACGCGCCGCC**ATGGACTACAAAGACGATGACGATAAAGCAAGAAGAGCATCTGTGCAT**-*ERα*-CAATTG

Underlines, double underline, and bold sequences are BsiWI/MfeI sites, kozak sequences, and FLAG/hexa-histidine (FH)-tag sequences, respectively. The position of *ERα* ORF from the 1st methionine to STOP codon is indicated in italic letters.

3) ERα or FoxA1 in baculovirus transfer vector pFastBac1 (pFastBac1-FH-ERα and pFastBac1-FH-FoxA1) for the recombinant protein expression in insect cells

AGATCT**ATGGACTACAAAGACGATGACGATAAAGCAAGAAGAGCATCTGTGCAT**-*ERα OR FoxA1*-GGATCC

Underlines and bold sequences are BglII/BamHI sites and FLAG/hexahistidine (FH)-tag sequences, respectively. The position of *ERα* or *FoxA1* ORF from the 1st methionine to STOP codon is indicated in italic letters.

4) pPVT1-DIV5-HMC2AT vector for the immobilized template assay

GAATTCGATATCAAGCTTATCGATACCGTCGACCTCGAGTGCATGCCGGGGATCC**TCCCTTTACATAGGACTACTTGGTAAATATTTACTGAATGAAGGAAAGCTGAAGTA**AGATCC**TCCCTTTACATAGGACTACTTGGTAAATATTTACTGAATGAAGGAAAGCTGAAGTA**AGATCC**TCCCTTTACATAGGACTACTTGGTAAATATTTACTGAATGAAGGAAAGCTGAAGTA**AGATCC**TCCCTTTACATAGGACTACTTGGTAAATATTTACTGAATGAAGGAAAGCTGAAGTA**AGATCC**TCCCTTTACATAGGACTACTTGGTAAATATTTACTGAATGAAGGAAAGCTGAAGTA**AGATCTGACTTCTAGG

The underlined sequences are the EcoRI site and the PstI site blunted by T4 DNA polymerase. The sequence downstream of the blunted PstI site is the sequence of the original pG5HMC2AT plasmid. The five FoxA1 binding sequences are shown in bold letters. For the mutant template, FoxA1 binding sequences were replaced to TCCCTTTACATAGGACTACTTGGT**G**A**GG**A**GG**TACTGAATGAAGGAAAGCTGAAGTA. The bold letters indicate the mutated sequences.

Antibodies used in this study

Antibody	Supplier	Catalog Number
Anti-FLAG M2	Sigma	F3165
Anti-ERα (D8H8)	CST Japan	8644
Anti-FoxA1	Abcam	ab23738
Anti-SRC1/NcoA1	Betyl	A300-343A
Anti-NcoA2/SRC2	Betyl	A300-346A
Anti-SRC-3/NcoA3 (5E11)	CST Japan	2126
Anti-MED1	Betyl	A300-793A
Anti- ESR1 (phospho S305)	Abnova	PAB12622

Cell culture medium

Mammalian cells were cultured at 37˚C in a humidified incubator in an atmosphere containing 5% CO2 except for cells cultured in Leibovitz's L-15 medium which were cultured without CO2. Insect cells were cultured by orbital shaking at 27˚C without CO2. Please refer to the following table for the medium used for each cell line. All cell lines and also established MDA-MB-453 cells inducibly expressing ERα (ERα/MDA-MB-453) were regularly maintained in the medium with phenol red. Note that the ERα/MDA-MB-453 were seeded with phenol red-free medium (asterisk in the table below) and then used for NGS (Fig 2, 3, and 4) and biochemical experiments (Fig 5). Luciferase assays with various cell lines were performed with each medium containing phenol red.

Mammalian cell line					
Cell Name	Medium	FBS (Gibco, 10270106)	Penicillin-Streptomycin (Fujifilm, 168-23191)	Insulin (SIGMA, 10516)	Other supplements
293T	DMEM (Nacalai, 08456-65)	10%	1%		
Hs578T	DMEM (Nacalai, 08456-65)	10%	1%	10ug/ml	
MCF7	DMEM (Nacalai, 08456-65)	10%	1%	10ug/ml	
YMB-1	RPMI (Nacalai, 30264-56)	10%	1%		
HCC1143	RPMI (Nacalai, 30264-56)	10%	1%		
HCC1806	RPMI (Nacalai, 30264-56)	10%	1%		
HCC1937	RPMI (Nacalai, 30264-56)	10%	1%		
HCC70	RPMI (Nacalai, 30264-56)	10%	1%		
HCC1395	RPMI (Nacalai, 30264-56)	10%	1%		
HCC38	RPMI (Nacalai, 30264-56)	10%	1%		
T-47D	RPMI (Nacalai, 30264-56)	10%	1%	10ug/ml	
BT549	RPMI (Nacalai, 30264-56)	10%	1%	10ug/ml	
BT-20	EMEM (WAKO, 051-07615)	10%	1%		
MDA-MB-453	McCoy's 5A (Cytiva, SH30200.01)	10%	1%		
MDA-MB-453*	McCoy's 5A without phenol red (Cytiva, SH30270.01)	10%	1%		

SUM159PT	Ham’s F-12 (WAKO, 087-08335)	5%	1%	5ug/ml	1ug/ml Hydrocortisone (Sigma, H0888-1G)
MCF 10A	Mammary Epithelial Cell Growth Medium Kit (Takara, C-21110)		1%		
MDA-MB-231	Leibovitz’s L-15 (WAKO, 128-06075)	10%	1%		
MDA-MB-468	Leibovitz’s L-15 (WAKO, 128-06075)	10%	1%		
MDA-MB-157	Leibovitz’s L-15 (WAKO, 128-06075)	10%	1%		
MDA-MB-436	Leibovitz’s L-15 (WAKO, 128-06075)	10%	1%	10ug/ml	
Insect cells					
Cell Name	Medium	Penicillin-Streptomycin (Fujifilm, 168-23191)			
Sf9	SF-900II SFM (Gibco, 10902096)	1%			
HighFive	SF-900II SFM (Gibco, 10902096)	1%			

RNA preparation and qPCR

Total RNA was purified by RNeasy (Qiagen) from cultured cells. Contaminated genomic DNA was removed by TURBO DNA-free kit (Thermo Fisher Scientific). PrimeScript RT master mix (TAKARA) was used for cDNA preparation. Quantitative PCR was performed with PowerUp SYBR green master mix (Thermo Fisher Scientific) with a pair of specific primers (see Supplemental Materials and Methods) and absolute amounts of transcript in samples were calculated from the standard reactions with concentration-known pTAC-2 plasmid introduced with each specific amplicon by TA PCR cloning kit (BDL).

Luciferase assay

Luciferase assays were performed as described [14] with minor modifications. Briefly, 1.5 X 10^5 cells were seeded onto 96-well plates with a medium containing phenol red without penicillin/streptomycin, cultured for one day, and transfected with 10 ng pGL3-GREB1-ERE3-fLuc, 0.04 ng pRL-SV (Promega), 24 ng pcDNA-3xF-ERα by using Lipofectamine 3000 (Invitrogen) according to the manufacturer’s instructions. After 24 hours,

10 nM 17β-estradiol (E2) was added for an additional 24 hours, and luciferase gene expression was measured by using Dual-Luciferase Reporter Assay System (Promega), and firefly luciferase activities were normalized against Renilla luciferase activities.

Recombinant protein purification

Baculoviruses expressing FH-ERα and FH-FoxA1 were prepared by the Bac-to-Bac baculovirus expression system (Thermo Fisher Scientific) with pFastBac1-FH-ERα and pFastBac1-FH-FoxA1 in Sf9 cells in according to manufacturer's instructions. High Five cells were infected with the prepared baculovirus to express recombinant proteins as described previously. The infected High Five cells were suspended in BC buffer [1] containing 0.1% NP40, 5 μM MG132 (Sigma), 0.5% protease inhibitor cocktail (Sigma), and 0.5 M NaCl for ERα or 0.3 M KCl for FoxA1, and sonicated to prepare whole cell lysate. After centrifugation, M2 agarose (Sigma) was mixed with the clear lysates and washed with lysis buffer. The bound proteins were eluted with lysis buffer containing 0.15 mg/ml 3xFLAG peptide (Sigma).

The nuclear extracts were prepared from MDA-MB-453 cells expressing WT or K303R ERα cultured in the medium without phenol red as described [14]. The nuclear extract was adjusted to BC300 containing 0.1% NP40, 5uM MG132, and 0.5% protease inhibitor cocktail, and then FH-ERα in the extract was purified as recombinant FH-ERα.

Lentivirus preparation

Lentiviruses expressing FLAG/His (FH)-tagged ERα were prepared by using the pAiLV-FH-ERα plasmid. pAiLV-FH-ERα was transfected into 293T cells with pMDLg/pRRE and pCMV-VSVG-RSV/Rev in the ratio of 2:1:1, with Lipofectamine 3000 (Invitrogen) according to the manufacturer's instructions. The medium was changed 18 hours after transfection, and the virus-containing medium was harvested twice after one-day incubation periods. The pooled virus suspension was filtered through a 0.45 um pore size filter and used for the infection.

Detailed ATAC-seq and ChIP-seq protocol

ATAC-seq and ChIP-seq were performed as described previously [2, 3] with the following modifications. One million MDA-MB-453 cells expressing ERα, which is maintained

in the regular medium, were seeded onto a 100 mm plate with the phenol red-free medium, cultured for 2 days, added with 2ug Dox and/or 1nM E2, further cultured for 2 days, and then used for the library preparation. To prepare ATAC-seq library, 5 x 10^4 cells per one sample were processed for nuclear pellets preparation, and tagmentation was performed with 2.5 ul of transposase of Tagment DNA Enzyme kit (Illumina) as described previously [2]. Prepared DNA fragments were cleaned with DNA Clean and concentrator-5 kit (Zymo Research) and added with adaptor and index sequences by PCR with NEBNext high-fidelity 2x PCR master mix (NEB), and then purified again with DNA Clean and concentrator-5 kit (Zymo Research). To prepare ChIP-seq library, 2.6x10^6 cells were seeded onto a 100 mm plate and treated with E2 and Dox as for ATAC-seq. Two plates were used for each immunoprecipitation. Fixation, nuclear pellet preparation, chromatin sharing, and immunoprecipitation were performed as described previously [3] with modifications. Briefly, chromatin was shared by Covaris E220 sonicator with the following setting; peak incident power=140, duty cycles=5%, cycles per burst=200, water level=8, time=10 min. Five microliters of anti-ESR1 antibody (Cell Signaling, 8644S) were used for one precipitation. Reverse crosslinked DNA was purified with AMPure XP beads (Agencourt) and DNA concentration was measured by Fluorometer (Quantus). ChIP-seq library was prepared from 2 ng of the purified DNA using SMARTer ThruPLEX DNA-seq Kit (TAKARA). The library was then purified again with AMPure XP beads (Agencourt). The concentration of the libraries was quantified using Fluorometer (Quantus) and KAPA library quantification kit (Roche), and the quality was checked by TapeStation D5000 (Agilent). The pooled libraries with unique index sequences were sequenced using MiSeq or NextSeq550 with pair-end 75 bp reads.

Data analysis

1) Peak calling of ATAC-seq data

1. Trimming adaptor sequences by skewer [4];

   Command line: skewer -f sanger -t 12 -m pe -x adaptor_sequences_files.fa --quiet -o ${name} ${name}_R1.fastq ${name}_R2.fastq

2. Quality check by fastqc;

   Command line: fastqc -o fastqc -f fastq --nogroup ${name}-trimmed-pair1.fastq ${name}-trimmed-pair2.fastq

3. Pre-alignment of reads to rCRSd to remove mitochondrial sequences by bowtie2 and samtools [5, 6];

   Command line:

   1. bowtie2 -p 12 -x rCRSd -k 1 -D 20 -R 3 -N 1 -L 20 -i S,1,0.50 -X 2000 --no-mixed --no-discordant -1 ${name}-trimmed-pair1.fastq -2 ${name}-trimmed-pair2.fastq -S ${name}_pre1.sam
   2. samtools sort -@ 12 -m 2G -n ${name}_pre1.sam > ${name}_pre1.sort.bam
   3. samtools view -@ 12 -S -f4 ${name}_pre1.sort.bam > ${name}_pre1.unmapped.sam
   4. cat ${name}_pre1.unmapped.sam | grep -v ^@ | awk 'NR%2==1 {print "@"$1"¥n"$10"¥n+¥n"$11}' > ${name}_pre1_r1.fastq
   5. cat ${name}_pre1.unmapped.sam | grep -v ^@ | awk 'NR%2==0 {print "@"$1"¥n"$10"¥n+¥n"$11}' > ${name}_pre1_r2.fastq

4. Pre-alignment of reads to human repeats to remove those sequences by bowtie2 and samtools [5, 6];

   Command line:

   1. bowtie2 -p 12 -x human_repeats -k 1 -D 20 -R 3 -N 1 -L 20 -i S,1,0.50 -X 2000 --no-mixed --no-discordant -1 ${name}_pre1_r1.fastq -2 ${name}_pre1_r2.fastq -S ${name}_pre2.sam
   2. samtools sort -@ 12 -m 2G -n ${name}_pre2.sam > ${name}_pre2.sort.bam
   3. samtools view -@ 12 -S -f4 ${name}_pre2.sort.bam > ${name}_pre2.unmapped.sam
   4. cat ${name}_pre2.unmapped.sam | grep -v ^@ | awk 'NR%2==1 {print "@"$1"¥n"$10"¥n+¥n"$11}' > ${name}_pre2_r1.fastq
   5. cat ${name}_pre2.unmapped.sam | grep -v ^@ | awk 'NR%2==0 {print "@"$1"¥n"$10"¥n+¥n"$11}' > ${name}_pre2_r2.fastq

5. Alignment of reads to hg38 by bowtie2 [6];

   Command line: bowtie2 -p 12 -x hg38 --very-sensitive -X 2000 --no-mixed --no-discordant -1 ${name}_pre2_r1.fastq -2 ${name}_pre2_r2.fastq -S ${name}_align.sam

6. Removing low quality reads by samtools [5];

   Command line:

   1. samtools view -bS -@ 12 ${name}_align.sam > ${name}_align.bam
   2. samtools sort -@ 12 ${name}_align.bam > ${name}_align.sort.temp.bam

3. samtools view -f 2 -q 10 -b -@ 12 -m 2G ${name}_align.sort.temp.bam > ${name}_align.sort.bam

7. Removing PCR duplicates by Picard (http://broadinstitute.github.io/picard/);
Command line: java -jar picard.jar MarkDuplicates I=${name}_align.sort.bam O=${name}.rmdup.bam M=metrics.file VALIDATION_STRINGENCY=LENIENT REMOVE_DUPLICATES=true

8. Peak calling by MACS2 [7];
Command line: macs2 callpeak -t ${name}_align.sort.bam -n ${name} -f BAM -g hs --shift -75 --extsize 150 --nomodel --call-summits --nolambda --keep-dup all -q 0.01 --outdir peak

2) Peak calling of ChIP-seq data

1. Quality filtering by FASTX-toolkit (http://hannonlab.cshl.edu/fastx_toolkit);
Command line: fastq_quality_filter -q 25 -p 90 -i ${name}_R1.fastq -o ${name}.qc.fastq

2. Alignment of reads to hg38 by bowtie2 [6];
Command line: bowtie2 -p 12 -x hg38 -U ${name}.qc.fastq -S ${name}.sam

3. Removing multi-mapped reads;
Command line:
grep -v "XS" ${name}.sam > ${name}.uq.sam
samtools view -Shb ${name}.uq.sam > ${name}.uq.bam
samtools sort -@ 12 -m 2G ${name}.uq.bam > ${name}.uq.sort.bam

4. Removing duplicated reads by Picard (http://broadinstitute.github.io/picard/);
Command line: java -jar picard.jar MarkDuplicates INPUT=${name}.uq.sort.bam OUTPUT=${name}.drm.bam METRICS_FILE=${name}.out.metrics AS=true REMOVE_DUPLICATES=true VALIDATION_STRINGENCY=LENIENT

5. Removing reads included in the black list by Bedtools [8];
Command line: intersectBed -abam ${name}.drm.bam -b hg38.blacklist.bed –v >${name}.drm.blalst.bam

6. Peak calling by MACS2 [7];
Command line; macs2 callpeak -t ${name}.drm.blalst.bam -n ${name}.drm.blalst -g hs --keep-dup auto

3) Other data analysis

To compare the similarity of multiple large data tables (ATAC-seq peak sets) statistically, principal component analysis (PCA), by which complex and multiple variables were reduced into the first two principal components, was performed by R Bioconductor package “Diffbind” (http://bioconductor.org/packages/release/bioc/html/DiffBind.html). In the figure, each data set was analyzed by PCA and plotted according to the first two principal components (PC); X-axis and Y-axis is showing the 1st and 2nd PC, respectively. The percentage means the proportion of how variances of data sets are explained by those PC. Also, differential accessible regions among multiple ATAC-seq read count data sets were analyzed and extracted and their correlation was calculated by differential binding affinity analysis (DBA) of “Diffbind” as well.

Condition- and clone-specific peaks were extracted by “mergepeaks” utility of Homer software [9] with options of “-d given”. Enrichment analysis of transcription factor binding motif at peak groups was performed by findMotifsGenome.pl utility of Homer software with the option of “hg38 -size 200”. The raw data of each enrichment analysis with raw peaks and condition-specific peaks of ATAC-seq, ChIP-seq, and ERα-ACR peaks were summarized in Supplemental Table 2. ERα-ACR peaks were selected by intersectBed utility of Bedtools [8] by intersecting ChIP-seq peaks by ATAC-seq peaks. Actual motif presence at each ChIP-seq peaks and ERα-ACR were counted by intersecting those peaks with bed files of each transcription factor in Homer software package. ERE half-site bed files were created from the sequence information of ERE half-site (Motif ID#M00959, MotifMap, http://motifmap.ics.uci.edu) by seq2profile.pl and scanMotifGenomeWide.pl of Homer software. Heatmap of peak intensity and average peak density profile were prepared by NGSplot [10].

For the comparison of enriched TFBM among WT, Y537S, and K303R peak groups (Fig 2C, 3C), remarkably enriched TFBMs are analyzed and represented as follows. Briefly, TFBMs enrichment score (-Log(p-value)) in the specific groups of Y537S/MDA-MB-453 and K303R/MDA-MB-453 were first compared with the corresponding groups of WT/MDA-MB-453 to calculate the LOG2FC. Then, if the calculated values of each TFBM met the criteria, the LOG2FC values with transcription factor names (gene symbol) and their family were represented in the figure. The criteria are as follows; LOG2FC≥3 and p-value difference of WT

and mutant ≥10^{-20} for Fig. 2C; LOG2FC≥2.3 and p-value difference of WT and mutant ≥10^{-20} for Fig. 3C Moreover, only transcription factors that were expressed in MDA-MB-453 cells identified from data of public RNA-seq analysis (GSE85870) and own scRNA-seq analysis of each clone (Supplemental Table 3), were used for the representation of all motif enrichment analysis (Fig. 2C, 3B, 3C, 4A, 4B, S2E). The correspondence between transcription factor name and raw motif name in Homer software was summarized in Supplemental Table 1.

Immobilized template assay

Immobilized template assays (ITA) were performed as described previously [11] with modifications. For ITAs with recombinant proteins, a biotinylated DNA fragment with tandem unique binding sequences for Gal4, ERα, and FoxA1 was amplified from the pG5HMC2AT, p4ERE-d53, and pPVT1-DIV5-HMC2AT, respectively, by PCR with a biotinylated M13R and M13F primer set. Two and a half microliters of the Dynabeads M280 streptavidin (Invitrogen) were immobilized with 100 ng template DNA and incubated with 50 µl of the blocking buffer (50 mM Tris-HCl pH7.9 at 4˚C, 100 mM KCl, 0.01% NP40, 10 mM DTT, 0.5 mM PMSF, 8 µg/ml single strand salmon sperm DNA [Thermo Fisher Scientific], 8 µg/ml polydI-dC [Sigma], 50 µg /ml BSA) at 30˚C for 15 min. Then, the 200 ng of recombinant protein in wash buffer (50 mM Tris-HCl pH7.9 at 4˚C, 100 mM KCl, 0.01% NP40, 0.5 mM DTT, 0.5 mM PMSF) was directly added to the beads in blocking buffer and mixed at 30˚C for 30 min with a continuous rotation. The beads were then washed three times at room temperature with 400 µl wash buffer. The template-bound proteins were finally eluted in 30 µl of SDS-PAGE sample buffer by boiling and subjected to SDS-PAGE followed by immunoblotting with specific antibodies to detect proteins as indicated in the figures. For ITA with the nuclear extract, 200 ng of the FoxA1 template were mixed with total 20 µl of the nuclear extracts including 50 µg total proteins derived from MDA-MB-453 expressing WT ERα and K303R ERα mutant which were adjusted to 100 mM KCl, instead of the recombinant proteins.

References

1. Nakadai T, Fukuda A, Shimada M, Nishimura K and Hisatake K (2015) The RNA binding complexes NF45-NF90 and NF45-NF110 associate dynamically with the c-fos gene and function as transcriptional coactivators. J Biol Chem 290:26832-45. doi: 10.1074/jbc.M115.688317

2. Corces MR, Trevino AE, Hamilton EG, Greenside PG, Sinnott-Armstrong NA, Vesuna S, Satpathy AT, Rubin AJ, Montine KS, Wu B, Kathiria A, Cho SW, Mumbach MR, Carter AC, Kasowski M, Orloff LA, Risca VI, Kundaje A, Khavari PA, Montine TJ, Greenleaf WJ and Chang HY (2017) An improved ATAC-seq protocol reduces background and enables interrogation of frozen tissues. Nat Methods 14:959-962. doi: 10.1038/nmeth.4396

3. Hinohara K, Wu HJ, Vigneau S, McDonald TO, Igarashi KJ, Yamamoto KN, Madsen T, Fassl A, Egri SB, Papanastasiou M, Ding L, Peluffo G, Cohen O, Kales SC, Lal-Nag M, Rai G, Maloney DJ, Jadhav A, Simeonov A, Wagle N, Brown M, Meissner A, Sicinski P, Jaffe JD, Jeselsohn R, Gimelbrant AA, Michor F and Polyak K (2018) KDM5 Histone Demethylase Activity Links Cellular Transcriptomic Heterogeneity to Therapeutic Resistance. Cancer Cell 34:939-953 e9. doi: 10.1016/j.ccell.2018.10.014

4. Jiang H, Lei R, Ding SW and Zhu S (2014) Skewer: a fast and accurate adapter trimmer for next-generation sequencing paired-end reads. BMC Bioinformatics 15:182. doi: 10.1186/1471-2105-15-182

5. Li H, Handsaker B, Wysoker A, Fennell T, Ruan J, Homer N, Marth G, Abecasis G, Durbin R and Genome Project Data Processing S (2009) The Sequence Alignment/Map format and SAMtools. Bioinformatics 25:2078-9. doi: 10.1093/bioinformatics/btp352

6. Langmead B and Salzberg SL (2012) Fast gapped-read alignment with Bowtie 2. Nat Methods 9:357-9. doi: 10.1038/nmeth.1923

7. Zhang Y, Liu T, Meyer CA, Eeckhoute J, Johnson DS, Bernstein BE, Nusbaum C, Myers RM, Brown M, Li W and Liu XS (2008) Model-based analysis of ChIP-Seq (MACS). Genome Biol 9:R137. doi: 10.1186/gb-2008-9-9-r137

8. Quinlan AR and Hall IM (2010) BEDTools: a flexible suite of utilities for comparing genomic features. Bioinformatics 26:841-2. doi: 10.1093/bioinformatics/btq033

9. Heinz S, Benner C, Spann N, Bertolino E, Lin YC, Laslo P, Cheng JX, Murre C, Singh H and Glass CK (2010) Simple combinations of lineage-determining transcription factors prime cis-regulatory elements required for macrophage and B cell identities. Mol Cell 38:576-89. doi: 10.1016/j.molcel.2010.05.004

10. Shen L, Shao N, Liu X and Nestler E (2014) ngs.plot: Quick mining and visualization of next-generation sequencing data by integrating genomic databases. BMC Genomics 15:284. doi: 10.1186/1471-2164-15-284

11. Chen W, Yang Q and Roeder RG (2009) Dynamic interactions and cooperative functions of PGC-1alpha and MED1 in TRalpha-mediated activation of the brown-fat-specific UCP-1 gene. Mol Cell 35:755-68. doi: 10.1016/j.molcel.2009.09.015
