# Supplementary material for: Estrogen receptor α K303R mutation reorganizes its binding to forkhead box protein A1 regions and induces chromatin opening
Source: Mol Biol Rep. 2022 Nov 27;50(2):1209–20. doi: 10.1007/s11033-022-08089-3 (PMC9889408; doi:10.1007/s11033-022-08089-3)
Supplement: Supplementary file 2 — Supplemental Figures (PDF 9973 kb) [file 11033_2022_8089_MOESM2_ESM.pdf]

Molecular Biology Reports

Estrogen receptor α K303R mutation reorganizes its binding to Forkhead box protein A1 regions and induces chromatin opening.

Tomoyoshi Nakadai[1], Liying Yang[1], Kohei Kumegawa[2], Reo Maruyama[1,2]

1 Project for Cancer Epigenomics, Cancer Institute, Japanese Foundation for Cancer Research, Tokyo, Japan.
2 Cancer Cell Diversity Project, NEXT-Ganken Program, Japanese Foundation for Cancer Research, Tokyo, Japan.

Corresponding author's contact information:
Tomoyoshi Nakadai
Project for Cancer Epigenomics
Cancer Institute, Japanese Foundation for Cancer Research
3-8-31, Ariake, Koto-ku, Tokyo, 135-8550, Japan
Tel: +81-3-3520-0111
E-mail: tomoyoshi.nakadai@jfcr.or.jp

Supplemental figure captions

Fig. S1 The BC cell lines tested for the luciferase assay, relates to Fig. 1. The BC cell lines tested for the luciferase assay was represented with various data and the summary of luciferase assay. The source, cell types, and the molecular subtypes are also shown [1-6]. The information of gene mutations, and relative mRNA and protein levels were obtained from DepMap (https://depmap.org/portal/). Each amino acid mutation position and type are also represented in according to the mutation nomenclature (https://www.hgmd.cf.ac.uk/docs/mut_nom.html). Note that ERBB2 mutation in MDA-MB-453 cells is a silent mutation. The absolute mRNA quantities in the actually tested cell lines were measured by own RT-qPCR analysis (see Materials and Methods). The results of luciferase assay were also summarized as types or *No ERE activity* meaning no firefly Luciferase signal detected. Abbreviations are: PB, primary breast; PE, pleural effusion; PeE, pericardial effusion; IDC, invasive ductal carcinoma; AC, anaplastic carcinoma; DC, ductal carcinoma; ASC, Acantholytic Squamous Cell Carcinoma; MC, metaplastic carcinoma; Ad, adenocarcinoma; FD, fibrocystic disease; Lu: Luminal; LuA: Luminal A; Ba, Basal; BaA, Basal A; BaB, Basal B; HER, HER2 positive; Me, Mesenchymal; Nor, Normal; CL, Claudin-low

Fig. S2 ATAC-seq analysis of Y537S/MDA-MB-453 and K303R/MDA-MB-453, relates to Fig. 2**. A.** ERα expression in each clone. Whole-cell extracts of each clone were subjected to SDS-PAGE followed by western blotting to detect ERα or βActin with specific antibodies as indicated. **B.** DBA of ATAC-seq peak data. Differential accessible region of each ATAC-seq peak data were analyzed by DBA and the correlation among the data was represented by the heatmap. Treatments of each sample are also indicated. **C.** Heatmap showing the signal intensity of WT ERα ATAC-seq peaks in each condition-specific group. ATAC-seq peaks of WT/MDA-MB-453 were classified into each condition-specific group (red boxes) and those corresponding genomic regions in other groups also shown in parallel. Peak intensities are plotted in a ±3kb window from summits. **D.** Average peak density profile of WT ERα in each condition-specific group (panel C). **E.** Enrichment of TFBMs at ATAC-seq peaks of Dox(+)/E2(-) and Dox(+)/E2(+)-specific groups of WT/MDA-MB-453. Log(p-value) of top 5 enriched TFBMs in each group and those corresponding values in other groups are represented with gene symbol, belonging family, and motif logo

Fig. S3 SDS-PAGE analysis of recombinant FoxA1 and ERα, relates to Fig. 5

Fig. S4 Expression levels of ERα-related cofactors. Expression levels of ERα-related cofactors in the various BC cell lines were obtained from Expression Atlas (https://www.ebi.ac.uk/gxa/home) and expression levels of each factor (TPM, transcripts per million) from multiple data sets were averaged and represented with a heatmap in parallel with the results of the luciferase assay. Because various ERα cofactors have been reported to contribute to E2-dependent and E2-independent ERα-driven transcription regulation [7-11]

Fig. S5 Relative expression levels of the Fox family members (TPM, transcripts per million) in MDA-MB-453 cells obtained from public RNA-seq (GSE85870) and own scRNA-seq analysis (unpublished data)

Fig. S6 Motif logos representing unique binding sequences of the various Fox family members stored in Homer software package

References

1. Neve RM, Chin K, Fridlyand J, Yeh J, Baehner FL, Fevr T, Clark L, Bayani N, Coppe JP, Tong F, Speed T, Spellman PT, DeVries S, Lapuk A, Wang NJ, Kuo WL, Stilwell JL, Pinkel D, Albertson DG, Waldman FM, McCormick F, Dickson RB, Johnson MD, Lippman M, Ethier S, Gazdar A and Gray JW (2006) A collection of breast cancer cell lines for the study of functionally distinct cancer subtypes. Cancer Cell 10:515-27. doi: 10.1016/j.ccr.2006.10.008

2. Charafe-Jauffret E, Ginestier C, Monville F, Finetti P, Adelaide J, Cervera N, Fekairi S, Xerri L, Jacquemier J, Birnbaum D and Bertucci F (2006) Gene expression profiling of breast cell lines identifies potential new basal markers. Oncogene 25:2273-84. doi: 10.1038/sj.onc.1209254

3. Sieuwerts AM, Kraan J, Bolt J, van der Spoel P, Elstrodt F, Schutte M, Martens JW, Gratama JW, Sleijfer S and Foekens JA (2009) Anti-epithelial cell adhesion molecule antibodies and the detection of circulating normal-like breast tumor cells. J Natl Cancer Inst 101:61-6. doi: 10.1093/jnci/djn419

4. Prat A, Parker JS, Karginova O, Fan C, Livasy C, Herschkowitz JI, He X and Perou CM (2010) Phenotypic and molecular characterization of the claudin-low intrinsic subtype of breast cancer. Breast Cancer Res 12:R68. doi: 10.1186/bcr2635

5. Subik K, Lee JF, Baxter L, Strzepek T, Costello D, Crowley P, Xing L, Hung MC, Bonfiglio T, Hicks DG and Tang P (2010) The Expression Patterns of ER, PR, HER2, CK5/6, EGFR, Ki-67 and AR by Immunohistochemical Analysis in Breast Cancer Cell Lines. Breast Cancer (Auckl) 4:35-41.

6. Dai X, Cheng H, Bai Z and Li J (2017) Breast Cancer Cell Line Classification and Its Relevance with Breast Tumor Subtyping. J Cancer 8:3131-3141. doi: 10.7150/jca.18457

7. Metivier R, Penot G, Hubner MR, Reid G, Brand H, Kos M and Gannon F (2003) Estrogen receptor-alpha directs ordered, cyclical, and combinatorial recruitment of cofactors on a natural target promoter. Cell 115:751-63. doi: 10.1016/s0092-8674(03)00934-6

8. McDonnell DP and Norris JD (2002) Connections and regulation of the human estrogen receptor. Science 296:1642-4. doi: 10.1126/science.1071884

9. Klinge CM, Jernigan SC, Mattingly KA, Risinger KE and Zhang J (2004) Estrogen response element-dependent regulation of transcriptional activation of estrogen receptors alpha and beta by coactivators and corepressors. J Mol Endocrinol 33:387-410. doi: 10.1677/jme.1.01541

10. Garcia-Pedrero JM, Kiskinis E, Parker MG and Belandia B (2006) The SWI/SNF chromatin remodeling subunit BAF57 is a critical regulator of estrogen receptor function in breast cancer cells. J Biol Chem 281:22656-64. doi: 10.1074/jbc.M602561200

11. Manavathi B, Samanthapudi VS and Gajulapalli VN (2014) Estrogen receptor coregulators and pioneer factors: the orchestrators of mammary gland cell fate and development. Front Cell Dev Biol 2:34. doi: 10.3389/fcell.2014.00034

Supplemental figures
Fig. S1

Cell Line	Sourse	Type	Molecular Subtypes						Data from DepMap													Absolute mRNA (log2((ymol/2ul cDNA)+1) (this study)			Luciferase assay results			Cell Line
									Cell line ID	Presense of mutation						Relative mRNA (log2(TPM+1))			Relative protein (log2(RPPA signal))									
			Neve et al., 2006	Charafe-Jauffret et al., 2006	Sieuwerts et al., 2009	Prat et al., 2010	Subik et al., 2010	Dai et al., 2017		*ESR1*	*ERBB2*	*PGR*	*TP53*	*BRCA1*	*BRCA2*	*ESR1*	*ERBB2*	*PGR*	ERa	HER2	PR	*ESR1*	*ERBB2*	*PGR*	Ectopic ERa-dependency	E2 responsiveness	Type	
MCF7	PE	IDC	Lu	Lu	Lu	-	Lu A	Lu A	ACH-000019							5.4	5.5	2.4	3.3	0.4	0.2	12.5	11.8	11.1	X	O	A	MCF7
T-47D	PE	IDC	Lu	Lu	Lu	-	NA	Lu A	ACH-000147				L194F			5.5	6.3	6.3	3.4	0.7	0.8	12.2	12.8	15.3	X	O	A	T-47D
MDA-MB-453	PeE	Ad	Lu	Lu	HER	-	-	HER	ACH-000910		L145L					0.0	8.0	0.0	0.6	1.7	-0.1	0.8	15.2	6.2	O	O	B	MDA-MB-453
YMB-1	PB	IDC	NA	NA	NA	NA	NA	NA	ACH-001249	NA	NA	NA	NA	NA	NA	NA	NA	NA	NA	NA	NA	5.5	14.3	3.3	O	O	B	YMB-1
BT-20	PB	IDC	BaA	Ba	Ba	-	Ba	BaA	ACH-000536				K132Q			2.0	6.1	0.0	0.8	0.5	-0.1	8.3	12.5	0.0	X	O	A	BT-20
HCC1143	PB	DC	BaA	NA	NA	-	NA	BaA	ACH-000374				NA			2.6	5.1	0.0	0.3	-0.4	0.0	6.8	12.2	0.0	No ERE activity			HCC1143
HCC1806	PB	ASC	NA	NA	NA	NA	NA	BaA	ACH-000624				T256fs			1.2	5.0	0.0	NA	NA	NA	8.9	12.3	0.0	O	X	C	HCC1806
HCC1937	PB	DC	BaA	Ba	Ba	-	NA	BaA	ACH-000223				R306*			2.2	5.6	0.0	0.4	0.0	0.1	9.3	12.3	0.0	No ERE activity			HCC1937
HCC70	PB	DC	BaA	NA	NA	-	Ba	BaA	ACH-000668				NA			1.4	5.4	0.0	-0.3	0.4	-0.2	0.0	12.8	0.0	No ERE activity			HCC70
MDA-MB-468	PE	Ad	BaA	NA	Ba	-	NA	BaA	ACH-000849		G152fs		R273H		M965I	1.2	4.6	0.0	0.0	-2.2	-0.1	7.2	10.6	0.0	No ERE activity			MDA-MB-468
HCC1395	PB	DC	NA	NA	NA	NA	NA	BaB	ACH-000699				R175H	R1751*	E1593*	0.0	4.2	0.0	-0.4	0.6	-0.5	6.3	13.8	1.5	No ERE activity			HCC1395
HCC38	PB	DC	BaB	Ba	NA	-	NA	BaB	ACH-000276				R273L		K644R	0.0	7.1	0.7	-0.2	1.2	-0.2	0.2	13.5	1.9	No ERE activity			HCC38
BT549	PB	IDC	BaB	NA	Nor	CL	NA	BaB	ACH-000288				R249S			0.0	4.1	0.1	NA	NA	NA	0.1	12.9	0.0	No ERE activity			BT549
SUM159PT	PB	AC	BaB	NA	Nor	CL	NA	BaB	ACH-001391			P359T	157_158insL			0.0	4.2	0.1	NA	NA	NA	0.8	11.8	0.7	O	X	C	SUM159PT
Hs578T	PB	IDC	BaB	Me	Nor	CL	NA	BaB	ACH-000148				V157F			0.1	4.7	0.0	0.2	-0.7	0.4	1.8	11.4	2.5	O	O	B	Hs578T
MDA-MB-157	PE	MC	BaB	Me	Nor	CL	NA	BaB	ACH-000621				APSWPLSS S88fs			0.2	4.5	0.2	-0.4	0.5	-0.1	2.2	11.4	6.9	No ERE activity			MDA-MB-157
MDA-MB-231	PE	Ad	BaB	Me	Nor	CL	Ba	BaB	ACH-000768				R280K			0.1	4.7	0.0	0.1	0.0	-0.1	0.9	11.3	0.0	No ERE activity			MDA-MB-231
MDA-MB-436	PE	Ad	BaB	NA	Nor	CL	HER	BaA	ACH-000573				E204fs	NA		0.1	4.0	0.0	0.1	-0.6	0.0	1.5	9.5	2.3	No ERE activity			MDA-MB-436
MCF 10A	PB	FD	BaB	NA	NA	NA	NA	NA	ACH-001357	NA	NA	NA	NA	NA	NA	NA	NA	NA	NA	NA	NA	0.9	10.3	0.0	No ERE activity			MCF 10A

Fig. S2

A

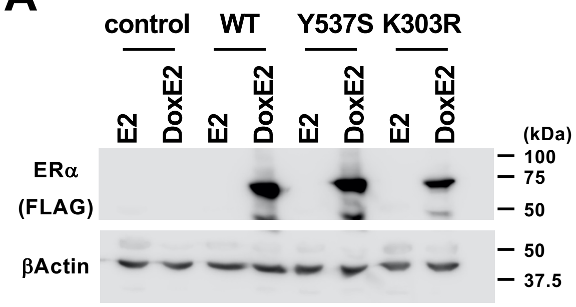

B

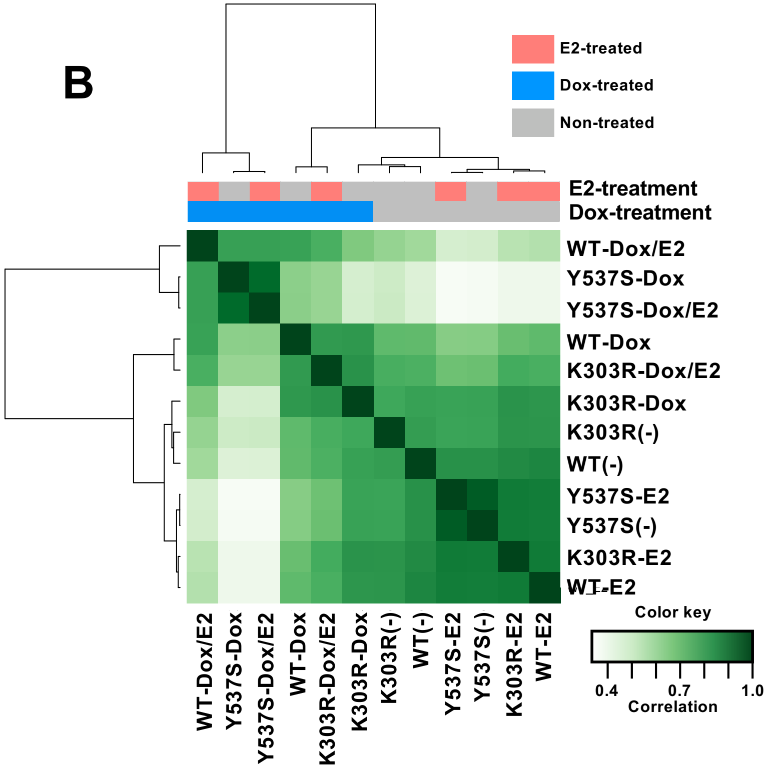

C

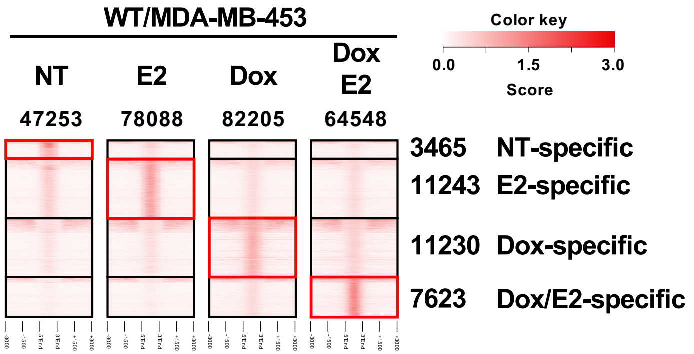

D

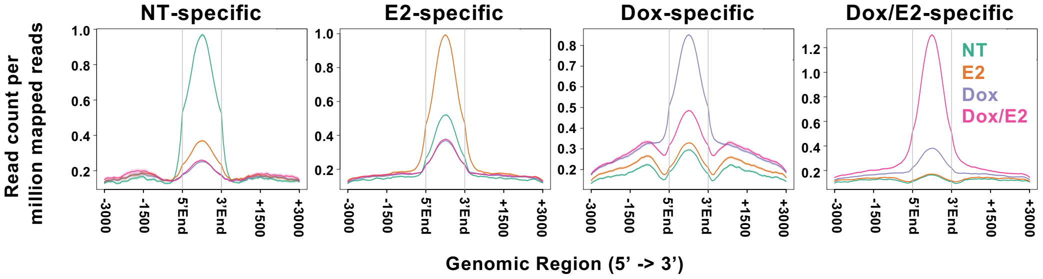

E

Motif Name	Motif	Transcription factor (Gene symbol)	Family	Log P-value no treat	Log P-value E2	Log P-value Dox	Log P-value Dox/E2
ERE(NR),IR3/MCF7-ERa-ChIP-Seq		*ESR1*	NHR	-1	0	-246	-1797
COUP-TFII(NR)/Artia-Nr2f2-ChIP-Seq		*NR2F2*	NHR	0	0	-48	-1110
EAR2(NR)/K562-NR2F6-ChIP-Seq		*NR2F6*	NHR	0	0	-14	-828
THRb(NR)/Liver-NR1A2-ChIP-Seq		*THRB*	NHR	-1	0	-81	-815
COUP-TFII(NR)/K562-NR2F1-ChIP-Seq		*NR2F1*	NHR	0	0	-20	-703
Fra1(bZIP)/BT549-Fra1-ChIP-Seq		*FOSL1*	AP1	-7	-97	-1614	-231
Fra2(bZIP)/Striatum-Fra2-ChIP-Seq		*FOSL2*	AP1	-6	-98	-1548	-228
JunB(bZIP)/DendriticCells-Junb-ChIP-Seq		*JUNB*	AP1	-8	-85	-1550	-225
BATF(bZIP)/Th17-BATF-ChIP-Seq		*BATF*	AP1	-9	-77	-1508	-224
Atf3(bZIP)/GBM-ATF3-ChIP-Seq		*ATF3*	CREB	-8	-88	-1471	-215

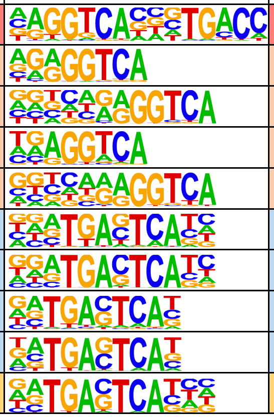

Fig. S3

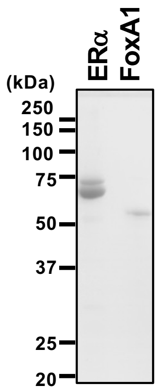

Fig. S4

	Luciferase assay results			TPM of each gene																							
							ERα Co-activator													ERα Co-repressor							
							LBD-binding co-activator											Co-activator binding to other than LBD		LBD-binding co-repressor						Co-repressor binding to other than LBD	
	Ectopic ERα-dependency	E2 responsiveness	Type	*ESR1*	*GATA3*	*FOXA1*	*MED1*	*NCOA1*	*NCOA2*	*NCOA3*	*PPARGC1A*	*PPARGC1B*	*PRC1*	*SRA1*	*DDX5*	*ERBB4*	*CITED*	*BAF57*	*CCND1*	*NCOR1*	*NCOR2*	*NRIP1*	*NR0B1*	*NR0B2*	*PHB2*	*BRCA1*	*UBE3A*
MCF7	X	O	A	56	876	182	18	14	33	586	0	3	175	47	864	1	36	18	541	32	37	40	0	0	228	17	20
T-47D	X	O	A	52	691	235	71	18	25	48	1	3	159	55	269	9	49	38	212	20	22	54	0	0	84	34	34
BT-20	X	O	A	3	68	56	44	29	27	137	0	2	242	30	208	0	25	15	191	40	49	90	0	0	342	21	32
YMB-1	O	O	B	40	123	207	47	20	42	71	0	7	172	26	510	23	65	30	614	34	97	17	NA	NA	710	22	57
Hs578T	O	O	B	0.1	5	2	50	18	31	55	0	1	14	61	342	0	185	3	322	48	97	13	0	0	211	23	27
MDA-MB-453	O	O	B	0	690	384	48	15	33	44	0	4	95	33	567	1	53	14	451	39	30	69	0	NA	301	12	51
HCC1806	O	X	C	1	44	24	31	5	9	67	0	4	50	15	291	0	63	10	381	41	81	33	3	0	220	18	55
SUM 159PT	O	X	C	N/A	N/A	N/A	68	13	16	46	NA	8	240	41	361	NA	88	31	442	32	103	16	NA	NA	448	46	38
HCC1143	No ERE activity			5	16	19	36	8	28	56	0	3	193	55	263	0	79	12	1644	32	26	172	0	NA	178	22	33
HCC1937	No ERE activity			4	26	25	58	14	32	73	0	7	77	61	336	0	85	11	488	39	57	59	0	2	309	40	26
HCC70	No ERE activity			2	69	50	34	18	61	42	1	1	113	35	221	0	24	8	121	26	55	40	0	1	168	25	30
MDA-MB-468	No ERE activity			2	44	33	37	33	23	48	0	5	115	49	233	0	173	18	253	24	67	103	0	NA	165	25	22
MDA-MB-157	No ERE activity			0.1	0.3	0.2	40	31	25	27	3	5	148	102	426	0	17	28	108	26	49	38	1	NA	174	21	33
MDA-MB-231	No ERE activity			0.1	10	0.1	46	12	7	23	1	1	160	43	265	0	78	23	543	36	38	7	0	NA	214	19	20
HCC1395	No ERE activity			0	0.7	0.2	41	8	18	66	1	6	133	46	258	0	35	14	216	32	56	34	37	0	560	8	24
HCC38	No ERE activity			0	35	10	94	21	33	55	0	16	146	48	469	0	104	24	618	34	74	107	3	1	175	9	45
BT549	No ERE activity			0	14	0	56	11	37	55	1	4	7	54	239	0	247	1	18	59	42	7	0	0	373	27	19
MDA-MB-436	No ERE activity			0	0.6	4	29	11	18	17	1	10	146	4	180	0	28	14	177	20	85	49	19	0	750	14	36
MCF 10A	No ERE activity			N/A	N/A	N/A	48	10	15	104	2	9	158	42	267	NA	28	26	171	44	23	19	NA	NA	397	32	36

Expression level (TPM)

MIN 0 MAX

Fig. S5

Gene Symbol	Relative TPM in RNA-seq Data (GSE85870)	Relative average TPM in our scRNA-seq Data
FOXA1	100	100
FOXM1	8	12
FOXK2	7	84
FOXP1	5	34
FOXO3	3	50
FOXK1	3	82
FOXO1	2	38
FOXH1	0	0
FOXA2	0	0
FOXA3	0	0
FOXD3	0	0
FOXF1	0	0
FOXL2	0	0

Fig. S6

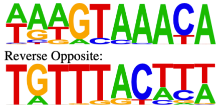

FoxA1(Forkhead)/LNCAP-FOXA1-ChIP-Seq(GSE27824)

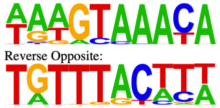

FoxA1(Forkhead)/MCF7-FOXA1-ChIP-Seq(GSE26831)

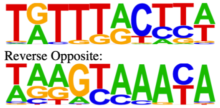

FoxM1(Forkhead)/MCF7-FOXM1-ChIP-Seq(GSE72977)

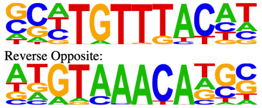

FoxK2(Forkhead)/U2OS-FOXK2-ChIP-Seq(E-MTAB-2204)
